# Supplementary material for: Modeling glioblastoma heterogeneity as a dynamic network of cell states
Source: Mol Syst Biol. 2021 Sep 16;17(9):e10105. doi: 10.15252/msb.202010105 (PMC8444284; doi:10.15252/msb.202010105)
Supplement: Supplementary file 6 — Source Data for Figure 5 [file MSB-17-e10105-s004.zip › Figure5A_sourcedata/GSEA_3017/hallmarks_stateE.GseaPreranked.1621934677158/HALLMARK_UV_RESPONSE_DN.html]

Details for gene set HALLMARK\_UV\_RESPONSE\_DN[GSEA]

|  || Dataset | state63017 |
| Phenotype | NoPhenotypeAvailable |
| Upregulated in class | na\_pos |
| GeneSet | HALLMARK\_UV\_RESPONSE\_DN |
| Enrichment Score (ES) | 0.67847574 |
| Normalized Enrichment Score (NES) | 3.1267939 |
| Nominal p-value | 0.0 |
| FDR q-value | 0.0 |
| FWER p-Value | 0.0 |
Table: GSEA Results Summary

  

Fig 1: Enrichment plot: HALLMARK\_UV\_RESPONSE\_DN      
 Profile of the Running ES Score & Positions of GeneSet Members on the Rank Ordered List

  

| PROBE | GENE SYMBOL | GENE\_TITLE | RANK IN GENE LIST | RANK METRIC SCORE | RUNNING ES | CORE ENRICHMENT || 1 | PMP22 |  |  | 0 | 1.393 | 0.1280 | Yes |
| 2 | IGFBP5 |  |  | 4 | 1.015 | 0.2179 | Yes |
| 3 | ANXA2 |  |  | 18 | 0.733 | 0.2712 | Yes |
| 4 | MAP1B |  |  | 22 | 0.715 | 0.3336 | Yes |
| 5 | GJA1 |  |  | 33 | 0.618 | 0.3796 | Yes |
| 6 | BDNF |  |  | 34 | 0.610 | 0.4356 | Yes |
| 7 | ID1 |  |  | 45 | 0.542 | 0.4746 | Yes |
| 8 | SERPINE1 |  |  | 52 | 0.526 | 0.5164 | Yes |
| 9 | F3 |  |  | 55 | 0.506 | 0.5608 | Yes |
| 10 | COL5A2 |  |  | 83 | 0.434 | 0.5714 | Yes |
| 11 | SIPA1L1 |  |  | 84 | 0.429 | 0.6108 | Yes |
| 12 | VLDLR |  |  | 87 | 0.419 | 0.6472 | Yes |
| 13 | PLPP3 |  |  | 139 | 0.342 | 0.6236 | Yes |
| 14 | IRS1 |  |  | 140 | 0.342 | 0.6550 | Yes |
| 15 | DDAH1 |  |  | 170 | 0.317 | 0.6528 | Yes |
| 16 | ATXN1 |  |  | 174 | 0.315 | 0.6785 | Yes |
| 17 | SDC2 |  |  | 226 | 0.280 | 0.6491 | No |
| 18 | INSIG1 |  |  | 252 | 0.261 | 0.6461 | No |
| 19 | PIK3R3 |  |  | 508 | -0.317 | 0.3998 | No |
| 20 | RBPMS |  |  | 519 | -0.320 | 0.4184 | No |
| 21 | MMP16 |  |  | 723 | -0.454 | 0.2408 | No |
Table: GSEA details [plain text format]

  

Fig 2: HALLMARK\_UV\_RESPONSE\_DN: Random ES distribution      
 Gene set null distribution of ES for **HALLMARK\_UV\_RESPONSE\_DN**
